# The Five‐Time Chair Stand Test Identifies Patients With Liver Cirrhosis at High Mortality Risk Overlooked by Conventional Sarcopenia Criteria

**DOI:** 10.1155/ijh/2748243

**Published:** 2026-09-06

**Authors:** Masafumi Haraguchi, Satoshi Miuma, Yasuhiko Nakao, Kosuke Takahashi, Masanori Fukushima, Ryu Sasaki, Hisamitsu Miyaaki

**Affiliations:** ^1^ Department of Gastroenterology and Hepatology, Nagasaki University Graduate School of Biomedical Sciences, Nagasaki, Japan, nagasaki-u.ac.jp

**Keywords:** five-time chair stand test, liver cirrhosis, physical performance, prognosis, sarcopenia

## Abstract

**Background:**

Sarcopenia is a major complication of liver cirrhosis (LC), but current Japan Society of Hepatology (JSH) criteria are based on muscle mass and strength and do not incorporate physical performance. We investigated whether the five‐time chair stand test (5T‐CST) identifies high‐risk patients not detected by conventional sarcopenia assessment.

**Methods:**

We retrospectively analyzed 241 patients with LC who underwent the 5T‐CST. Low physical performance was defined as a 5T‐CST time ≥ 12 s. Patients were classified according to their handgrip strength and physical performance, with focus on those with normal handgrip strength.

**Results:**

Longer 5T‐CST duration was associated with lower handgrip strength and sarcopenia, but not low skeletal muscle mass index. Among 151 patients with normal handgrip strength and survival data, 27 (17.9%) had low physical performance. This subgroup had preserved muscle mass but more ascites and hepatic reserve impairment and lower survival (mortality, 29.6% vs. 12.1%; log‐rank *p* = 0.007). In a principal analysis restricted to this subgroup, adjusted for mALBI grade and hepatocellular carcinoma (HCC), low physical performance independently predicted mortality (hazard ratio, 4.70; 95% CI, 1.94–11.37; *p* < 0.001). In the overall cohort (*n* = 237), it remained predictive after adjustment for age, sex, mALBI grade, HCC, and sarcopenia (hazard ratio, 2.74; 95% CI, 1.45–5.16; *p* = 0.002).

**Conclusions:**

Among patients with LC and normal handgrip strength, the 5T‐CST identified a subgroup at a high risk of mortality. Adding this simple test to sarcopenia assessment may help identify patients otherwise missed by handgrip strength alone.

## 1. Introduction

Sarcopenia is a major complication of liver cirrhosis (LC) and is directly associated with impaired quality of life, accelerated progression to liver failure, and poor survival [1–3]. Since the Japan Society of Hepatology (JSH) established diagnostic criteria for sarcopenia in liver disease in 2016, assessment of skeletal muscle mass index (SMI) and handgrip strength has been incorporated into routine clinical practice in Japan and has become an essential component of patient management and prognostic evaluation [4–7].

However, the current JSH criteria focus on muscle mass and strength and do not incorporate physical performance as a diagnostic component [3, 8]. Physical performance is increasingly recognized as a distinct dimension that reflects the severity and prognosis of sarcopenia beyond muscle quantity alone [9]. The 2025 update of the Asian Working Group for Sarcopenia (AWGS 2025) adopted a framework that separates decline in physical performance from the core diagnosis of sarcopenia and redefines it as an outcome [10], reflecting a broader shift toward viewing physical performance as an independent indicator of severity and prognosis. Consequently, diagnostic criteria based solely on muscle mass and strength may fail to identify patients whose physical function is already impaired.

Among the available measures of physical performance, gait speed and the Short Physical Performance Battery (SPPB) are widely used but require dedicated space and time, thereby limiting their feasibility in busy outpatient settings [4, 11]. In contrast, the five‐time chair stand test (5T‐CST), a component of the SPPB, can be performed rapidly using only a single chair and reflects lower limb strength and dynamic balance [12–14]. Although the 5T‐CST has been associated with survival in patients with LC [15], these studies have primarily focused on its prognostic value. It remains unclear what additional information physical performance assessment provides beyond the muscle mass‐based and strength‐based JSH criteria, particularly whether it can identify high‐risk patients who are classified as having normal muscle strength by conventional strength assessment.

We therefore hypothesized that the 5T‐CST captures prognostically relevant impairment that is not reflected by muscle mass or handgrip strength. In a cohort of patients with LC managed according to the JSH criteria, this study is aimed at (1) characterizing the discordance between physical performance, assessed using the 5T‐CST, and the components of sarcopenia (muscle mass and strength) and (2) determining the clinical and prognostic significance of low physical performance among patients with normal handgrip strength.

## 2. Materials and Methods

### 2.1. Study Participants and Design

This retrospective study included 294 patients with LC who were admitted to Nagasaki University Hospital between June 2021 and April 2025. We excluded 10 patients with cancers other than liver cancer, active infections, or psychiatric disorders. Of the remaining 284 eligible patients, 43 did not undergo the 5T‐CST. Consequently, 241 patients who completed the test were included in the final analysis (Figure S1). Baseline characteristics of the 43 patients who did not undergo the 5T‐CST were compared with those of the 241 analyzed patients and are reported in Table S1. The study protocol was approved by the Institutional Review Board of the Nagasaki University Ethics Committee (Approval No. 25051502). The study was conducted in accordance with the Declaration of Helsinki. As this was a retrospective study, the requirement for informed consent was waived, and an opt‐out option was provided on the hospital′s website to allow patients to decline participation.

Clinical characteristics and laboratory data were retrospectively collected from medical records. The etiology of liver disease was determined based on clinical findings, laboratory testing, imaging, and histological results. LC was diagnosed using clinical findings, laboratory data, imaging modalities (ultrasonography, computed tomography [CT], or magnetic resonance imaging [MRI]), and/or histological evidence. Hepatic reserve was evaluated using the Child–Pugh (CP) grade and the modified albumin‐bilirubin (mALBI) grade. The mALBI grade [16] was calculated using a previously reported formula based on serum albumin and total bilirubin levels, and patients were categorized into four grades (1, 2a, 2b, and 3). Ascites was graded according to the International Club of Ascites classification (Grade 0, none; Grade 1, detectable only by imaging; Grade 2, moderate symmetrical abdominal distension; Grade 3, marked abdominal distension). No patient had overt hepatic encephalopathy (West Haven grade ≥ 2) at the time of the 5T‐CST and body composition assessment, confirmed by medical record review; covert hepatic encephalopathy was not systematically assessed in this retrospective cohort. The 5T‐CST, handgrip strength measurement, and body composition assessment were performed during a clinically stable period of hospitalization.

### 2.2. Evaluation of Skeletal Muscle, Muscle Strength, and Sarcopenia

Sarcopenia was diagnosed according to the criteria established by the JSH [3, 7], which define sarcopenia as the presence of both low handgrip strength and a low SMI. Handgrip strength was assessed in the standing position using a Smedley handgrip dynamometer (TTM, Tokyo, Japan). As recommended by the AWGS 2019 criteria [4], two measurements were obtained from each hand, and the highest value was used for analysis. Low strength grip was defined as < 28 kg for males and < 18 kg for females. SMI was calculated using a direct segmental multifrequency bioelectrical impedance analyzer (InBody 770; InBody Japan, Tokyo, Japan), which measures the impedance of the four limbs and the trunk separately at multiple frequencies. Measurements were performed in the standing position over approximately 1 min in the fasting state. Appendicular skeletal muscle mass was derived as the sum of the lean soft tissue mass of the four limbs obtained directly from the segmental measurements; no external prediction equation was applied. SMI was calculated as appendicular skeletal muscle mass divided by height squared (kilogram per square meter). Low SMI was defined as < 7.0 kg/m^2^ for males and < 5.7 kg/m^2^ for females.

### 2.3. Assessment of Physical Performance

Physical performance was evaluated using the 5T‐CST [12–14]. Patients were instructed to stand up from a chair and sit down five times as quickly as possible with their arms folded across their chest. The time required to complete five repetitions was recorded. According to the AWGS 2019 criteria, low physical performance was defined as a 5 T‐CST time of ≥ 12 s [4]. Additionally, to examine the association between physical performance and clinical outcomes, patients were stratified into four categories based on established clinical cutoffs: ≤ 9 s, 10–11 s, 12–15 s, and ≥ 16 s. These thresholds were based on the AWGS 2019 cutoff for low physical performance (12 s) and a previously reported threshold associated with increased mortality in patients with cirrhosis (15 s) [4, 15].

### 2.4. Statistical Analysis

Continuous variables are expressed as median (interquartile ranges [IQRs]) or mean (standard deviations), as appropriate, and categorical variables as numbers and percentages. Differences between two groups were evaluated using the Wilcoxon rank‐sum test for continuous variables and the chi‐squared test or Fisher′s exact test, as appropriate, for categorical variables. Comparisons among the four groups defined by handgrip strength and 5T‐CST status were performed using the Kruskal–Wallis test for continuous variables and the chi‐squared test or Fisher′s exact test for categorical variables. Trends in the prevalence of low handgrip strength and sarcopenia across 5T‐CST categories were evaluated using the Jonckheere–Terpstra test.

Cumulative survival was estimated using the Kaplan–Meier method, and between‐group differences were compared using the log‐rank test. The log‐rank test for trend was used to evaluate stepwise survival trends across ordered categories.

A Cox proportional hazards model was used to identify independent predictors of overall survival. To ensure clinical relevance and minimize overfitting, covariates for the multivariable model were selected a priori based on established clinical knowledge and previous literature rather than statistical significance alone. Age and sex were included as fundamental demographic variables, and the mALBI grade and the presence of hepatocellular carcinoma (HCC) were incorporated because they are well‐established prognostic determinants in patients with LC. mALBI grade was coded as a binary variable (Grade 3 vs. Grades 1/2a/2b) in all models. Conventional sarcopenia status (JSH‐defined) was included as an additional covariate in the principal overall‐cohort model; sensitivity analyses in which handgrip strength and SMI were entered as separate covariates, and a formal test for interaction between handgrip strength status and physical performance status, are reported in Table S2, together with events per variable (EPV) for each model. Overall survival was defined as the time from the date of the 5T‐CST to death from any cause; patients who underwent liver transplantation during follow‐up (*n* = 24) were censored at the date of transplantation, and a competing risk analysis treating transplantation as a competing event is reported in Table S3. Low physical performance (5T‐CST ≥ 12 s) was then added to the base model as the primary variable of interest to evaluate its independent prognostic value. Multicollinearity was assessed using the variance inflation factor (VIF).

All statistical tests were two‐sided, and a *p* value < 0.05 was considered statistically significant. Statistical analyses were performed using R Version 4.6.1 (R Foundation for Statistical Computing, Vienna, Austria) and GraphPad Prism Version 9 (GraphPad Software, San Diego, California, United States).

## 3. Results

### 3.1. Patient Characteristics

The baseline characteristics of the 241 patients included in the final analysis are summarized in Table 1. The median age was 71 years, and 164 patients (68.0%) were male. The median body mass index (BMI) was 23.5 kg/m^2^. The etiologies of LC were metabolic dysfunction‐associated steatotic liver disease (MASLD; *n* = 72), alcohol‐related liver disease (*n* = 62), hepatitis C virus infection (*n* = 37), hepatitis B virus infection (*n* = 32), and other causes (*n* = 38). CP grade data were available for 237 patients (four patients had missing data). Of these, 147 (62.0%) were classified as Grade A, 58 (24.5%) as Grade B, and 32 (13.5%) as Grade C. According to the mALBI grade, 53, 49, 93, and 46 patients were categorized as Grades 1, 2a, 2b, and 3, respectively. Any ascites (Grade ≥ 1) was present in 72 of 235 patients with available data (30.6%; Grade 0/1/2/3, 163/48/23/1; missing in 6 patients).

**Table 1:** Characteristics of participants.

Characteristics	All (*n* = 241)
Age (years)	71.0 [62.0–77.0]
Sex, men (%)	164 (68.0)
BMI (kg/m^2^)	23.5 [21.0–26.0]
Etiology: MASLD/alcohol/HCV/HBV/AIH/PBC/others/unknown	72/62/37/32/12/6/13/7
Child–Pugh grade, A/B/C^a^	147/58/32
mALBI grade, 1/2a/2b/3	53/49/93/46
Ascites, any (%)^b^	72 (30.6)
Plt (× 10^4^/*μ*L)	12.3 [8.5–17.7]
PT (%)	81 [66–91]
Alb (g/dL)	3.4 [2.9–3.9]
Total bilirubin (mg/dL)	1.0 [0.6–1.5]
Cre (mg/dL)	0.81 [0.68–0.95]
Type IV collagen 7S (ng/mL)	7.0 [4.7–11.6]
SMI (kg/m^2^), male/female	7.1 [6.4–7.8]/6.0 [5.4–6.6]
Low SMI (%)	105 (43.6)
Handgrip strength (kg), male/female	30.6 [26.3–35.7]/19.3 [16.8–21.9]
Low handgrip strength (%)	86 (35.7)
Sarcopenia (%)	52 (21.6)
5T‐CST (s)	9.0 [8.0–12.0]
Low physical performance (%)	66 (27.4)
Classification of 5T‐CST, ≤ 9/10–11/12–15/≥ 16 s, *n* (%)	122 (50.6)/53 (22.0)/42 (17.4)/24 (10.0)
Median follow‐up (days)	236 [84–548]
Deaths during follow‐up, *n* (%)	46 (19.1)

*Note:* Data are presented as median [interquartile range] or *n* (%).

Abbreviations: AIH, autoimmune hepatitis; Alb, albumin; BMI, body mass index; Cre, creatinine; HBV, hepatitis B virus; HCV, hepatitis C virus; mALBI, modified albumin‐bilirubin; MASLD, metabolic dysfunction‐associated steatotic liver disease; PBC, primary biliary cholangitis; Plt, platelet; PT, prothrombin time; SMI, skeletal muscle mass index.

^a^Child–Pugh grade was missing in four patients.

^b^Ascites was graded according to the International Club of Ascites classification; missing in six patients.

The median handgrip strength was 30.6 kg in males and 19.3 kg in females. Based on the JSH criteria, 86 patients (35.7%) had low handgrip strength, 105 (43.6%) had a low SMI, and 52 (21.6%) were diagnosed with sarcopenia. The median 5T‐CST time was 9 s, and 66 patients (27.4%) had low physical performance (5T‐CST ≥ 12 s) as shown in Table 1; patients were also stratified into four categories based on 5T‐CST duration: ≤ 9 s (*n* = 122), 10–11 s (*n* = 53), 12–15 s (*n* = 42), and ≥ 16 seconds (*n* = 24). During a median follow‐up of 236 days, 46 patients died.

### 3.2. Dissociation Between Physical Performance and Skeletal Muscle Mass

**Figure 1:**
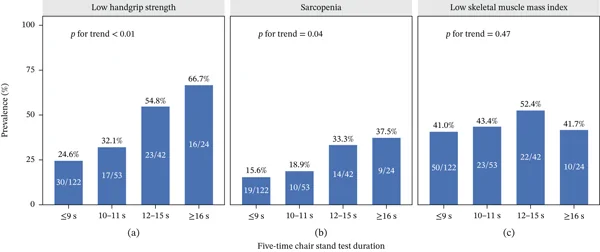
Dissociation between physical performance and skeletal muscle mass across 5T‐CST categories. (a) Low handgrip strength, (b) sarcopenia, and (c) low skeletal muscle mass index. The percentages of patients with the respective conditions are shown above the bars, and the absolute numbers of patients are indicated within the bars. *p* values were calculated using the Jonckheere–Terpstra test. SMI, skeletal muscle mass index.

Across the four 5T‐CST categories, the Jonckheere–Terpstra test showed a significant increasing trend in the prevalence of low handgrip strength (*p* < 0.01) (Figure 1a) and sarcopenia (*p* = 0.04) (Figure 1b) with longer 5T‐CST duration. In contrast, no significant trend was observed for the prevalence of low SMI (*p* = 0.47) (Figure 1c). These findings indicate that impaired physical performance was associated with reduced muscle strength, but not reduced muscle mass, suggesting that muscle mass assessment alone does not capture impaired physical performance.

### 3.3. Discordance Between Handgrip Strength and Physical Performance

Given that physical performance was associated with muscle strength, we next examined whether these measures identified the same patients. Patients were classified into four groups according to handgrip strength and 5T‐CST status (Table 2). Among the 151 patients with normal handgrip strength and available survival data, 27 (17.9%) had low physical performance, although handgrip strength and 5T‐CST status were significantly associated overall (*χ* ^2^, *p* < 0.001).

**Table 2:** Patient characteristics according to handgrip strength and 5T‐CST status.

Characteristics	Grip‐normal/chair‐normal (*n* = 128)	Grip‐normal/chair‐low (*n* = 27)	Grip‐low/chair‐normal (*n* = 47)	Grip‐low/chair‐low (*n* = 39)	*p* value
Age (years)	69.0 [62.0–74.0]	72.0 [59.5–76.5]	74.0 [61.5–81.0]	75.0 [64.0–83.0]	0.008
Sex, men (%)	91 (71.1)	16 (59.3)	32 (68.1)	25 (64.1)	0.618
Child–Pugh grade, A/B/C^b^	83/26/15	12/7/8	31/15/1	21/10/8	0.003^a^
Ascites (%)	32 (25.8)	13 (50.0)	11 (23.9)	16 (41.0)	0.033
mALBI grade, 1/2a/2b/3	38/30/38/22	4/4/11/8	6/8/28/5	5/7/16/11	0.009
Low SMI (%)	44 (34.4)	9 (33.3)	29 (61.7)	23 (59.0)	0.001
Mortality (%)^b^	15 (12.1)	8 (29.6)	11 (23.4)	12 (30.8)	0.016

*Note:* Data are presented as median [interquartile range] or *n* (%). *p* values were calculated using the Kruskal–Wallis test for continuous variables and the chi‐squared test or Fisher′s exact test for categorical variables, as appropriate.

Abbreviations: mALBI, modified albumin‐bilirubin; SMI, skeletal muscle mass index.

^a^Fisher′s exact test, Child–Pugh Grade C versus Grade A/B.

^b^Child–Pugh grade and survival follow‐up data were missing in the same four patients within this group; these four patients were excluded from the survival‐related analyses described in the text (*n* = 151 with survival data among grip‐normal patients overall).

Among patients with normal handgrip strength, the prevalence of low SMI was similar regardless of physical performance status (33.3% in the chair‐low subgroup vs. 34.4% in the chair‐normal subgroup), indicating that this discordant subgroup could not be identified by either handgrip strength or muscle mass assessment. Across the four groups, the prevalence of ascites and the distributions of CP grade and mALBI grade differed significantly (overall *p* = 0.033, *p* = 0.003, and *p* = 0.009, respectively), with the highest prevalence of ascites and of CP Grade C observed among patients with normal handgrip strength but low physical performance. Thus, among patients with preserved handgrip strength, low physical performance was associated with features of advanced liver disease that were not captured by the muscle mass and strength components of sarcopenia. Overall mortality also differed significantly across the four groups (*p* = 0.016).

### 3.4. Prognostic Significance of Physical Performance Among Patients With Normal Handgrip Strength

**Figure 2:**
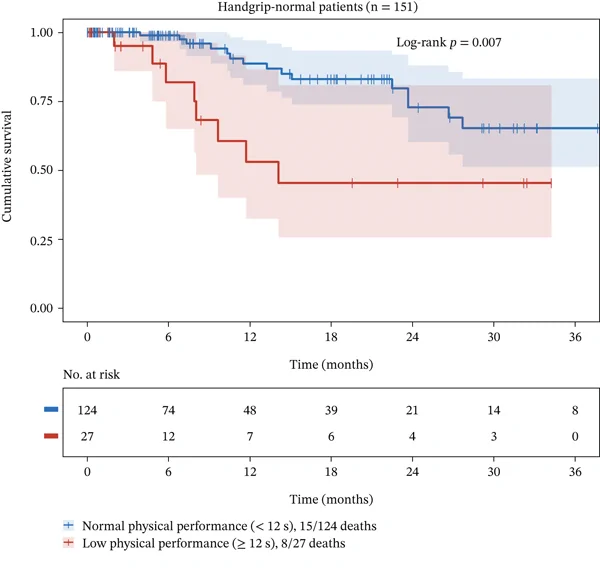
Kaplan–Meier survival curves according to physical performance status among patients with normal handgrip strength. Cumulative survival is shown for patients with normal handgrip strength, stratified by 5T‐CST status (low physical performance, ≥ 12 s, *n* = 27; normal physical performance, < 12 s, *n* = 124). The number of deaths in each group (15/124 and 8/27, respectively) is shown in the legend, and 95% confidence bands are displayed for each curve. The *p* value was calculated using the log‐rank test.

We next examined whether low physical performance had prognostic significance in the subgroup with normal handgrip strength. Among the 151 patients with normal handgrip strength and available survival data, Kaplan–Meier analysis demonstrated that those with low physical performance (≥ 12 s; *n* = 27) had significantly lower cumulative survival than those with normal physical performance (*n* = 124) (log‐rank test, *p* = 0.007) (Figure 2). Crude mortality was 29.6% (8/27) in the low physical performance subgroup compared with 12.1% (15/124) in the normal physical performance subgroup. These findings indicate that, even among patients with normal handgrip strength, the 5T‐CST identified a subgroup at substantially higher risk of death.

Consistent with these findings, Kaplan–Meier analysis of the overall cohort also showed significantly lower survival in patients with low physical performance and a stepwise decline in survival across the four 5T‐CST categories (Figure S2).

### 3.5. Independent Predictors of Survival

To determine whether low physical performance predicts mortality independently of hepatic reserve, HCC, and conventional sarcopenia status, we performed univariate and multivariable Cox proportional hazards analyses of overall survival (Table 3). In the overall cohort (*n* = 237, 46 deaths), univariate analysis showed that mALBI Grade 3 and low physical performance (5T‐CST ≥ 12 s) were significantly associated with poor survival. In the multivariable model, which included the a priori selected variables of age, sex, mALBI grade, HCC, and conventional sarcopenia status, male sex (hazard ratio [HR], 3.49; 95% CI, 1.51–8.10; *p* = 0.004), mALBI Grade 3 (HR, 7.16; 95% CI, 3.28–15.60; *p* < 0.001), HCC (HR, 4.59; 95% CI, 1.47–14.34; *p* = 0.009), and low physical performance (HR, 2.74; 95% CI, 1.45–5.16; *p* = 0.002) were independent predictors of mortality, whereas sarcopenia itself was not significant (HR, 0.77; *p* = 0.50) (Table 3, Panel A). A likelihood ratio test confirmed that adding low physical performance to a model already containing sarcopenia status significantly improved model fit (*χ* ^2^ = 9.15, *p* = 0.002).

**Table 3:** Cox proportional hazards models for overall survival. Overall cohort (*n* = 237; 46 deaths).

Variables	Unadjusted HR	95% CI	*p* value	Adjusted HR	95% CI	*p* value
Age	1.01	0.99–1.04	0.330	1.00	0.97–1.04	0.809
Sex, men	3.03	1.35–6.80	0.007	3.56	1.53–8.29	0.003
mALBI Grade 3 (vs. 1/2a/2b)	3.60	1.85–7.00	< 0.001	7.09	3.25–15.5	< 0.001
HCC, present	3.73	1.33–10.4	0.012	4.68	1.50–14.6	0.008
Sarcopenia, present	1.00	0.52–1.93	1.000	0.77	0.37–1.62	0.50
Low physical performance	1.89	1.05–3.40	0.033	2.74	1.45–5.16	0.002

*Note:* The multivariable model included age, sex, mALBI grade (Grade 3 vs. 1/2a/2b), the presence of HCC, conventional sarcopenia status, and low physical performance (5T‐CST ≥ 12 s), selected a priori. All variance inflation factors were < 1.5. *n* = 237, events = 46. EPV = 46/6 = 7.7. Concordance index = 0.749.

As a principal analysis addressing patients of primary clinical interest, we next restricted the multivariable model to the 151 patients with normal handgrip strength (23 deaths), adjusting for mALBI grade and HCC. In this subgroup, low physical performance was a strong independent predictor of mortality (HR, 4.70; 95% CI, 1.94–11.37; *p* < 0.001), with mALBI Grade 3 and HCC also associated with mortality (Table 4).

**Table 4:** Patients with normal handgrip strength (*n* = 151; 23 deaths)—principal analysis.

Variables	Unadjusted HR	95% CI	*p* value	Adjusted HR	95% CI	*p* value
mALBI Grade 3 (vs. 1/2a/2b)	2.34	0.79–6.93	0.124	3.01	0.99–9.12	0.051
HCC, present	4.11	0.95–17.8	0.058	6.60	1.49–29.2	0.013
Low physical performance	3.08	1.30–7.28	0.010	4.70	1.94–11.4	< 0.001

*Note:* The multivariable model included mALBI grade (Grade 3 vs. 1/2a/2b) and the presence of HCC, restricted to patients classified as normal by conventional handgrip strength assessment. *n* = 151, events = 23. Concordance index = 0.720. All variance inflation factors were < 1.1. This model addresses patients of primary clinical interest and is presented as our principal multivariable analysis.

Abbreviations: CI, confidence interval; HCC, hepatocellular carcinoma.HR, hazard ratio; mALBI, modified albumin‐bilirubin.

In sensitivity analyses, when handgrip strength and SMI were entered as separate covariates instead of the composite sarcopenia variable, the association between low physical performance and mortality was attenuated (HR, 1.99; 95% CI, 0.97–4.05; *p* = 0.060), consistent with the limited EPV in this model (EPV = 6.6). A formal interaction analysis showed a significant interaction between handgrip strength status and physical performance status (*p* = 0.006), indicating that the prognostic effect of low physical performance was concentrated among patients with normal handgrip strength (Table S2). This pattern is consistent with the results of the principal subgroup analysis described above.

Across these analyses, low physical performance consistently retained independent prognostic value after adjustment for hepatic reserve, HCC, and conventional sarcopenia status, indicating that its prognostic information was not explained by liver function, the presence of liver cancer, or conventional sarcopenia measures alone.

## 4. Discussion

In this study of patients with LC managed according to the JSH criteria, low physical performance assessed using the 5T‐CST identified a subgroup at high risk of mortality that was not detected using the muscle mass and strength components of sarcopenia. Among patients with normal handgrip strength, those with low physical performance had significantly poorer survival than those with preserved performance, and this subgroup was characterized by preserved muscle mass but advanced liver disease. Furthermore, low physical performance remained an independent predictor of mortality after adjustment for age, sex, hepatic reserve, HCC, and conventional sarcopenia status, and this association was even stronger when restricted to patients with normal handgrip strength. These findings indicate that the 5T‐CST captures prognostically relevant impairment that is overlooked by conventional sarcopenia assessment.

A key observation was the dissociation between physical performance and skeletal muscle mass. Although longer 5T‐CST duration was associated with a higher prevalence of low handgrip strength and sarcopenia, no corresponding trend was observed for low SMI. This discrepancy suggests that physical performance reflects a dimension of muscle function that is not captured by muscle quantity alone. Unlike handgrip strength, which measures a localized and absolute muscle force, the 5T‐CST requires repeated elevation of body weight and therefore reflects lower limb power relative to body mass, as well as dynamic balance and motor coordination [17]. Lower limb function is closely linked to mobility and activities of daily living, and its decline is a recognized precursor to falls and reduced physical activity [18–20]. This pattern, in which physical function rather than muscle mass predicts adverse outcomes, has also been reported in other populations, including in older patients with heart failure and community‐dwelling older adults [21, 22]. Moreover, among patients with preserved handgrip strength, low physical performance was associated with a higher prevalence of ascites and more advanced hepatic reserve impairment, suggesting that the 5T‐CST also reflects systemic decompensation beyond muscle mass and strength.

These findings are consistent with the conceptual shift proposed in the AWGS 2025 update, which repositions physical performance from a diagnostic component of sarcopenia to an indicator of disease severity and prognosis [10]. Our results provide empirical support for this framework in patients with LC managed according to the JSH criteria. Specifically, physical performance identified high‐risk patients who would be classified as normal using criteria based solely on muscle mass and strength, suggesting that incorporating physical performance assessment may address an important gap in the current sarcopenia assessment for patients with LC.

From a practical perspective, the 5T‐CST is particularly suited for routine clinical practice. Unlike gait speed and the SPPB, which require dedicated space and time, the 5T‐CST can be performed rapidly using only a single chair [11]. Because it identified high‐risk patients undetected by muscle mass and strength assessment, the 5T‐CST may serve as a simple screening tool to identify patients who may benefit from closer monitoring; whether nutritional or rehabilitative intervention improves outcomes in this subgroup was not evaluated in the present study and should be addressed in prospective intervention trials [23].

Although previous studies have shown that the 5T‐CST predicts survival in patients with LC [15, 24], they primarily established its prognostic value. Our study extends these findings by demonstrating the incremental diagnostic value of physical performance within the established JSH framework, particularly its ability to identify high‐risk patients with normal handgrip strength. Furthermore, the independent association between low physical performance and mortality after adjustment for hepatic reserve, HCC, and conventional sarcopenia status indicates that its prognostic value is not explained solely by liver function, the presence of liver cancer, or conventional sarcopenia measures.

This study has several limitations. First, it was a single‐center retrospective study, which may limit the generalizability of the findings. Second, the 5T‐CST was not performed in all eligible patients because it was either omitted from routine assessment or could not be completed owing to severe mobility limitations, introducing the potential for selection bias; comparison of tested and nontested patients suggested that this bias, if present, likely resulted in conservative estimates (Table S1). Third, the subgroup of primary interest—patients with normal handgrip strength but low physical performance—was relatively small, and these findings should be regarded as hypothesis‐generating. Fourth, detailed information on the causes of death was unavailable, limiting our ability to distinguish liver‐related and non–liver‐related mortality; a competing risk analysis treating liver transplantation as a competing event yielded consistent results (Table S3). Finally, the limited number of events restricted the number of covariates included in the multivariable model (EPV as low as 6.6 in sensitivity analyses), and mALBI grade was coded as a binary variable (Grade 3 vs. Grades 1/2a/2b) rather than as a full ordinal variable, and some estimates should therefore be interpreted with caution. Covert hepatic encephalopathy and the timing of variceal assessment relative to the 5T‐CST were not systematically evaluated in this retrospective cohort. Prospective multicenter studies are needed to validate these findings and to establish the optimal role of physical performance assessment in the management of patients with LC.

In conclusion, among patients with LC and normal handgrip strength, the 5T‐CST identified a subgroup at increased risk of mortality that would not have been detected by conventional sarcopenia assessment. Incorporating this simple physical performance test into sarcopenia assessment may help identify patients who would otherwise be missed by handgrip strength assessment alone, supporting its use alongside handgrip strength and muscle mass assessment for risk stratification in patients with LC.

## Author Contributions

**Masafumi Haraguchi:** conceptualization, methodology, formal analysis, investigation, writing – original draft, writing – review and editing. **Satoshi Miuma:** conceptualization, methodology, formal analysis, writing – review and editing. **Yasuhiko Nakao:** investigation, data curation. **Kosuke Takahashi:** investigation, data curation. **Masanori Fukushima:** investigation, data curation. **Ryu Sasaki:** investigation, data curation.

**Hisamitsu Miyaaki:** conceptualization, supervision.

## Funding

No funding was received for this manuscript.

## Disclosure

The authors reviewed and edited the content as needed and take full responsibility for the content of the published article.

## Ethics Statement

The study protocol was approved by the Institutional Review Board of the Nagasaki University Ethics Committee (Approval No. 25051502). The study was conducted in accordance with the Declaration of Helsinki. As this was a retrospective study, the requirement for informed consent was waived, and an opt‐out option was provided on the hospital′s website to allow patients to decline participation.

## Conflicts of Interest

The authors declare no conflicts of interest.

## Supporting Information

Additional supporting information can be found online in the Supporting Information section.

## Supporting information


**Supporting Information 1** Figure S1. Flowchart of the study population. The flowchart illustrates the patient selection and exclusion process.

**Supporting Information 2** Figure S2. Kaplan–Meier survival curves for the overall cohort based on 5T‐CST duration. (a) Comparison between two groups using the 12‐s cutoff among all 237 patients with available survival data, and (b) comparison across four categories (≤ 9, 10–11, 12–15, and ≥ 16 s). *p* values were calculated using the log‐rank test and log‐rank test for trend.

**Supporting Information 3** Table S1. Comparison of patients who did and did not undergo the five‐time chair stand test. Baseline characteristics and outcomes are compared between the 43 patients who did not undergo the 5T‐CST and the 241 patients who completed it. Table S2. Sensitivity analyses addressing the independent contribution of low physical performance beyond handgrip strength and skeletal muscle mass. Results are shown for models in which handgrip strength and skeletal muscle mass index were entered as separate covariates and for a formal test of interaction between handgrip strength status and physical performance status. Table S3. Competing risk analysis of overall mortality, with liver transplantation treated as a competing event. Cumulative incidence functions, cause‐specific Cox models, and Fine–Gray subdistribution hazard models are presented for the overall cohort and for the subgroup with normal handgrip strength.

**Supporting Information 4**  

## Data Availability

The data supporting the findings of this study are available from the corresponding author upon reasonable request.

## References

[1] European Association for the Study of the Liver , EASL Clinical Practice Guidelines on Nutrition in Chronic Liver Disease, Journal of Hepatology. (2019) 70, no. 1, 172–193, 10.1016/j.jhep.2018.06.024, 30144956. 30144956 PMC6657019

[2] Kawaguchi T. , Kawaguchi A. , Hashida R. , Nakano D. , Tsutsumi T. , Kawaguchi M. , Koya S. , Hirota K. , Tomita M. , Tsuchihashi J. , Narao H. , Matsuse H. , Hiraoka K. , Ejima K. , Iwami S. , and Yoshio S. , Resistance Exercise in Combination With Aerobic Exercise Reduces the Incidence of Serious Events in Patients With Liver Cirrhosis: A Meta-Analysis of Randomized Controlled Trials, Journal of Gastroenterology. (2024) 59, no. 3, 216–228, 10.1007/s00535-023-02060-0, 38159112. 38159112

[3] Yoshiji H. , Nagoshi S. , Akahane T. , Asaoka Y. , Ueno Y. , Ogawa K. , Kawaguchi T. , Kurosaki M. , Sakaida I. , Shimizu M. , Taniai M. , Terai S. , Nishikawa H. , Hiasa Y. , Hidaka H. , Miwa H. , Chayama K. , Enomoto N. , Shimosegawa T. , Takehara T. , and Koike K. , Evidence-Based Clinical Practice Guidelines for Liver Cirrhosis 2020, Hepatology Research. (2021) 51, no. 7, 725–749, 10.1111/hepr.13678, 34228859. 34228859

[4] Chen L. K. , Woo J. , Assantachai P. , Auyeung T. W. , Chou M. Y. , Iijima K. , Jang H. C. , Kang L. , Kim M. , Kim S. , Kojima T. , Kuzuya M. , Lee J. S. W. , Lee S. Y. , Lee W. J. , Lee Y. , Liang C. K. , Lim J. Y. , Lim W. S. , Peng L. N. , Sugimoto K. , Tanaka T. , Won C. W. , Yamada M. , Zhang T. , Akishita M. , and Arai H. , Asian Working Group for Sarcopenia: 2019 Consensus Update on Sarcopenia Diagnosis and Treatment, Journal of the American Medical Directors Association. (2020) 21, no. 3, 300–307.e2, 10.1016/j.jamda.2019.12.012. 32033882

[5] Cruz-Jentoft A. J. , Bahat G. , Bauer J. , Boirie Y. , Bruyère O. , Cederholm T. , Cooper C. , Landi F. , Rolland Y. , Sayer A. A. , Schneider S. M. , Sieber C. C. , Topinkova E. , Vandewoude M. , Visser M. , Zamboni M. , and Writing Group for the European Working Group on Sarcopenia in Older People 2 (EWGSOP2), and the Extended Group for EWGSOP2 , Sarcopenia: Revised European Consensus on Definition and Diagnosis, Ageing and Health. (2019) 48, no. 1, 16–31, 10.1093/ageing/afy169, 30312372. PMC6322506 30312372

[6] Lai J. C. , Tandon P. , Bernal W. , Tapper E. B. , Ekong U. , Dasarathy S. , and Carey E. J. , Malnutrition, Frailty, and Sarcopenia in Patients With Cirrhosis: 2021 Practice Guidance by the American Association for the Study of Liver Diseases, Hepatology. (2021) 74, no. 3, 1611–1644, 10.1002/hep.32049, 34233031. 34233031 PMC9134787

[7] Nishikawa H. , Shiraki M. , Hiramatsu A. , Moriya K. , Hino K. , and Nishiguchi S. , Japan Society of Hepatology Guidelines for Sarcopenia in Liver Disease (1st Edition): Recommendation From the Working Group for Creation of Sarcopenia Assessment Criteria, Hepatology Research. (2016) 46, no. 10, 951–963, 10.1111/hepr.12774, 27481650. 27481650

[8] Nishikawa H. , Shiraki M. , Hiramatsu A. , Hara N. , Moriya K. , Hino K. , and Koike K. , Reduced Handgrip Strength Predicts Poorer Survival in Chronic Liver Diseases: A Large Multicenter Study in Japan, Hepatology Research. (2021) 51, no. 9, 957–967, 10.1111/hepr.13679, 34057800. 34057800

[9] Tandon P. , Montano-Loza A. J. , Lai J. C. , Dasarathy S. , and Merli M. , Sarcopenia and Frailty in Decompensated Cirrhosis, Journal of Hepatology. (2021) 75, S147–S162, 10.1016/j.jhep.2021.01.025, 34039486. 34039486 PMC9125684

[10] Chen L.-K. , Hsiao F.-Y. , Akishita M. , Assantachai P. , Lee W.-J. , Lim W. S. , Muangpaisan W. , Kim M. , Merchant R. A. , Peng L. N. , Tan M. P. , Won C. W. , Yamada M. , Woo J. , and Arai H. , A Focus Shift From Sarcopenia to Muscle Health in the Asian Working Group for Sarcopenia 2025 Consensus Update, Nature Aging. (2025) 5, no. 11, 2164–2175, 10.1038/s43587-025-01004-y, 41188603. 41188603

[11] Welch S. A. , Ward R. E. , Beauchamp M. K. , Leveille S. G. , Travison T. , and Bean J. F. , The Short Physical Performance Battery (SPPB): A Quick and Useful Tool for Fall Risk Stratification Among Older Primary Care Patients, Journal of the American Medical Directors Association. (2021) 22, no. 8, 1646–1651, 10.1016/j.jamda.2020.09.038, 33191134. 33191134 PMC8113335

[12] Albalwi A. A. and Alharbi A. A. , Optimal Procedure and Characteristics in Using Five Times Sit to Stand Test Among Older Adults: A Systematic Review, Medicine, Baltimore. (2023) 102, no. 26, e34160, 10.1097/MD.0000000000034160, 37390277. 37390277 PMC10313281

[13] Duncan R. P. , Leddy A. L. , and Earhart G. M. , Five Times Sit-to-Stand Test Performance in Parkinson′s Disease, Archives of Physical Medicine and Rehabilitation. (2011) 92, no. 9, 1431–1436, 10.1016/j.apmr.2011.04.008, 21878213. 21878213 PMC3250986

[14] Park T. S. and Shin M.-J. , Comprehensive Assessment of Lower Limb Function and Muscle Strength in Sarcopenia: Insights From the Sit-to-Stand Test, Annals of Geriatric Medicine and Research. (2024) 28, no. 1, 1–8, 10.4235/agmr.23.0205, 38325818. 38325818 PMC10982452

[15] Nguyen V. V. , Wang S. , Whitlock R. , Xu C. , Taneja S. , Singh S. , Abraldes J. G. , Burak K. , Bailey R. J. , Grab J. D. , Lai J. C. , and Tandon P. , A Chair-Stand Time of Greater Than 15 Seconds Is Associated With an Increased Risk of Death and Hospitalization in Cirrhosis, Canadian Liver Journal. (2023) 6, no. 3, 358–362, 10.3138/canlivj-2022-0021, 38020188. 38020188 PMC10652985

[16] Hiraoka A. , Michitaka K. , Kumada T. , Izumi N. , Kadoya M. , Kokudo N. , Kubo S. , Matsuyama Y. , Nakashima O. , Sakamoto M. , Takayama T. , Kokudo T. , Kashiwabara K. , Kudo M. , and The Liver Cancer Study Group of Japan , Validation and Potential of Albumin-Bilirubin Grade and Prognostication in a Nationwide Survey of 46, 681 Hepatocellular Carcinoma Patients in Japan: The Need for a More Detailed Evaluation of Hepatic Function, Liver Cancer. (2017) 6, no. 4, 325–336, 10.1159/000479984, 29234636. 29234636 PMC5704689

[17] Losa-Reyna J. , Alcazar J. , Carnicero J. , Alfaro-Acha A. , Castillo-Gallego C. , Rosado-Artalejo C. , Rodríguez-Mañas L. , Ara I. , and García-García F. J. , Impact of Relative Muscle Power on Hospitalization and All-Cause Mortality in Older Adults, Journals of Gerontology, Series A: Biological Sciences and Medical Sciences. (2022) 77, no. 4, 781–789, 10.1093/gerona/glab230, 34407184. 34407184

[18] Aamann L. , Dam G. , Borre M. , Drljevic-Nielsen A. , Overgaard K. , Andersen H. , Vilstrup H. , and Aagaard N. K. , Resistance Training Increases Muscle Strength and Muscle Size in Patients With Liver Cirrhosis, Clinical Gastroenterology and Hepatology. (2020) 18, no. 5, 1179–1187.e6, 10.1016/j.cgh.2019.07.058, 31394282. 31394282

[19] Hatta K. , Ikebe K. , Mihara Y. , Gondo Y. , Kamide K. , Masui Y. , Sugimoto K. , Matsuda K. I. , Fukutake M. , Kabayama M. , Shintani A. , Ishizaki T. , Arai Y. , Rakugi H. , and Maeda Y. , Lack of Posterior Occlusal Support Predicts the Reduction in Walking Speed in 80-Year-Old Japanese Adults: A 3-Year Prospective Cohort Study With Propensity Score Analysis by the SONIC Study Group, Gerodontology. (2019) 36, no. 2, 156–162, 10.1111/ger.12393, 30724390. 30724390

[20] Osaka T. , Hamaguchi M. , Hashimoto Y. , Ushigome E. , Tanaka M. , Yamazaki M. , and Fukui M. , Decreased the Creatinine to Cystatin C Ratio Is a Surrogate Marker of Sarcopenia in Patients With Type 2 Diabetes, Diabetes Research and Clinical Practicer. (2018) 139, 52–58, 10.1016/j.diabres.2018.02.025, 29496508. 29496508

[21] Honma S. , Katano S. , Yano T. , Nagaoka R. , Habaguchi A. , Numazawa R. , Ohori K. , Kouzu H. , Katayose M. , Yoshioka N. , Furuhashi M. , and Hashimoto A. , Determinant of Functional Independence in Older Patients With Heart Failure: Physical Function vs. muscle mass, Archives of Gerontology and Geriatrics. (2025) 137, 105933, 10.1016/j.archger.2025.105933, 40543452. 40543452

[22] Zanker J. , Scott D. , Alajlouni D. , Kirk B. , Bird S. , DeBruin D. , Vogrin S. , Bliuc D. , Tran T. , Cawthon P. , Duque G. , and Center J. R. , Mortality, Falls and Slow Walking Speed Are Predicted by Different Muscle Strength and Physical Performance Measures in Women and Men, Archives of Gerontology and Geriatrics. (2023) 114, 105084, 10.1016/j.archger.2023.105084, 37290229. 37290229

[23] Tandon P. , Ismond K. P. , Riess K. , Duarte-Rojo A. , Al-Judaibi B. , Dunn M. A. , Holman J. , Howes N. , Haykowsky M. J. , Josbeno D. A. , and McNeely M. , Exercise in Cirrhosis: Translating Evidence and Experience to Practice, Journal of Hepatology. (2018) 69, no. 5, 1164–1177, 10.1016/j.jhep.2018.06.017, 29964066. 29964066

[24] De Monte S. , Altmann P. , Pichlmeier S. , Leicht H. B. , Stuhlreiter S. , Brandl R. , Reiter F. P. , Hahn S. , Benoit C. , Geier A. , and Plauth M. , Mortality Prediction by Bedside Rectus Femoris Muscle Ultrasound for Sarcopenia Diagnosis in Liver Cirrhosis, United European Gastroenterology Journal. (2025) 13, no. 10, 1936–1945, 10.1002/ueg2.70114, 41045465. 41045465 PMC12704570

