# Supplementary material for: The Five‐Time Chair Stand Test Identifies Patients With Liver Cirrhosis at High Mortality Risk Overlooked by Conventional Sarcopenia Criteria
Source: Int J Hepatol. 2026 Sep 6;2026:2748243. doi: 10.1155/ijh/2748243 (PMC13547947; doi:10.1155/ijh/2748243)
Supplement: Supplementary file 1 [file IJH-2026-2748243-s003.docx]

**Supplementary Table S1. Comparison of patients who did and did not undergo the five-time chair stand test**

| **Characteristics** | **Not tested (n = 43)** | **Tested (n = 241)** | **p-value** |
| --- | --- | --- | --- |
| Age, years | 69.0 [59.0–74.5] | 71.0 [62.0–77.0] | 0.28 |
| Sex, men (%) | 34 (79.1) | 164 (68.0) | 0.21 |
| BMI, kg/m² | 24.9 [21.6–26.8] | 23.5 [21.0–26.0] | 0.20 |
| Albumin, g/dL | 3.2 [2.6–3.5] | 3.4 [2.9–3.9] | 0.008 |
| Total bilirubin, mg/dL | 1.0 [0.7–1.6] | 1.0 [0.6–1.5] | 0.65 |
| Child-Pugh grade, A/B/C | 20/13/9 | 147/58/32 | 0.23† |
| mALBI grade 3 (%) | 11 (25.6) | 46 (19.1) | 0.31 |
| Ascites (%) | 16 (38.1) | 72 (30.6) | 0.37 |
| Handgrip strength, kg | 24.6 [19.9–30.8] | 27.0 [21.0–33.1] | 0.29 |
| Low handgrip strength (%) | 22 (51.2) | 86 (35.7) | 0.062 |
| Follow-up, days | 141.5 [47.2–274.5] | 236.0 [84.0–548.0] | 0.059 |
| Mortality (%) | 12 (28.6) | 46 (19.4) | 0.21 |

*Data are presented as median [interquartile range] or number (percentage).*

*p-values were calculated using the Mann-Whitney U test for continuous variables and Fisher's exact test for categorical variables.*

*†p-value for Child-Pugh grade C versus grade A/B.*

*Child-Pugh grade and survival data were missing in one patient in the not-tested group and in four patients in the tested group; percentages were calculated using available data.*

*BMI, body mass index; mALBI, modified albumin-bilirubin.*

**Supplementary Table S2. Sensitivity analyses addressing the independent contribution of low physical performance beyond handgrip strength and skeletal muscle mass (overall cohort, n = 237; 46 deaths)**

| **Variable** | **HR** | **95% CI** | **p-value** |
| --- | --- | --- | --- |
| ***A. Handgrip strength and SMI entered separately (adjusted for age, sex, mALBI grade, and HCC)*** |  |  |  |
| Low handgrip strength | 1.88 | 0.85–4.19 | 0.12 |
| Low SMI | 0.64 | 0.32–1.29 | 0.21 |
| Low physical performance | 1.99 | 0.97–4.05 | 0.060 |
| ***B. Interaction between handgrip strength status and physical performance status (adjusted for age, sex, mALBI grade, and HCC)*** |  |  |  |
| Handgrip strength, low | 3.23 | 1.39–7.50 | 0.006 |
| Physical performance, low | 5.43 | 2.24–13.15 | <0.001 |
| Handgrip strength (low) × Physical performance (low) | 0.18 | 0.06–0.62 | 0.006 |

*Model A: events per variable (EPV) = 46/7 = 6.6. This model tests whether low physical performance remains associated with mortality when handgrip strength and skeletal muscle mass index (SMI) are entered as separate covariates rather than as a composite sarcopenia variable (cf. Table 3, Panel A).*

*Model B: a likelihood ratio test comparing the model with and without the interaction term was significant (χ2 = 7.01, 1 df, p = 0.008), indicating that the prognostic effect of low physical performance was substantially stronger among patients with normal handgrip strength than among those with low handgrip strength. This finding is consistent with the results of the principal subgroup analysis restricted to patients with normal handgrip strength (Table 3, Panel B).*

*Low SMI and low handgrip strength were defined according to the Japan Society of Hepatology criteria. Low physical performance was defined as a five-time chair stand test time ≥ 12 seconds. mALBI grade was coded as a binary variable (grade 3 vs. grades 1/2a/2b).*

*CI, confidence interval; HCC, hepatocellular carcinoma; HR, hazard ratio; mALBI, modified albumin-bilirubin; SMI, skeletal muscle mass index.*

**Supplementary Table S3. Competing risk analysis of overall mortality, with liver transplantation treated as a competing event**

**A. Cumulative incidence of death and liver transplantation**

| **Outcome / group** | **180 days** | **365 days** | **730 days** |
| --- | --- | --- | --- |
| **Death** |  |  |  |
| Normal performance (n = 171) | 3.1% | 13.6% | 29.4% |
| Low performance (n = 66) | 13.1% | 31.3% | 41.7% |
| **Liver transplantation** |  |  |  |
| Normal performance | 6.9% | 11.2% | 11.2% |
| Low performance | 12.0% | 12.0% | 14.8% |

*Gray's test for cumulative incidence of death: p = 0.035. Gray's test for cumulative incidence of liver transplantation: p = 0.29.*

**B. Overall cohort (n = 237; 46 deaths, 24 transplantations)**

| **Variable** | **HR** | **95% CI** | **p-value** |
| --- | --- | --- | --- |
| ***Cause-specific Cox model for death (transplantation censored)*** |  |  |  |
| Age | 1.00 | 0.97–1.04 | 0.81 |
| Sex, men | 3.56 | 1.53–8.27 | 0.003 |
| mALBI grade 3 | 7.09 | 3.25–15.49 | <0.001 |
| HCC, present | 4.68 | 1.50–14.60 | 0.008 |
| Low physical performance | 2.60 | 1.40–4.82 | 0.002 |
| ***Cause-specific Cox model for liver transplantation (death censored)*** |  |  |  |
| Age | 0.97 | 0.94–1.00 | 0.069 |
| Sex, men | 0.85 | 0.36–2.01 | 0.70 |
| mALBI grade 3 | 7.75 | 2.93–20.53 | <0.001 |
| HCC, present | 0.46 | 0.16–1.30 | 0.14 |
| Low physical performance | 1.05 | 0.45–2.46 | 0.91 |
| ***Fine–Gray subdistribution hazard model for death*** |  |  |  |
| Age | 1.02 | 0.98–1.05 | 0.33 |
| Sex, men | 3.07 | 1.34–7.03 | 0.008 |
| mALBI grade 3 | 3.97 | 1.78–8.84 | <0.001 |
| HCC, present | 5.21 | 1.67–16.20 | 0.004 |
| Low physical performance | 2.28 | 1.24–4.20 | 0.008 |
| ***Fine–Gray subdistribution hazard model for death, additionally adjusted for sarcopenia*** |  |  |  |
| Age | 1.02 | 0.98–1.06 | 0.28 |
| Sex, men | 3.03 | 1.32–6.94 | 0.009 |
| mALBI grade 3 | 3.96 | 1.77–8.84 | <0.001 |
| HCC, present | 5.14 | 1.65–15.94 | 0.005 |
| Sarcopenia | 0.82 | 0.38–1.77 | 0.62 |
| Low physical performance | 2.37 | 1.27–4.45 | 0.007 |

**C. Patients with normal handgrip strength (n = 151; 23 deaths, 18 transplantations)**

| **Variable** | **HR** | **95% CI** | **p-value** |
| --- | --- | --- | --- |
| ***Cause-specific Cox model for death*** |  |  |  |
| mALBI grade 3 | 3.02 | 1.00–9.14 | 0.051 |
| HCC, present | 6.58 | 1.48–29.15 | 0.013 |
| Low physical performance | 4.71 | 1.94–11.40 | <0.001 |
| ***Fine–Gray subdistribution hazard model for death*** |  |  |  |
| mALBI grade 3 | 1.20 | 0.40–3.60 | 0.74 |
| HCC, present | 8.01 | 1.79–35.78 | 0.006 |
| Low physical performance | 4.27 | 1.76–10.39 | 0.001 |

*Cumulative incidence of death within the normal-handgrip subgroup was 1.1% vs 14.6% at 180 days, 10.1% vs 37.4% at 365 days, and 23.8% vs 43.4% at 730 days for patients with normal versus low physical performance (Gray's test, p = 0.020).*

*Cumulative incidence functions were estimated using the Aalen–Johansen estimator and compared using Gray's test. Cause-specific hazard ratios were estimated using Cox proportional hazards models in which the competing event was treated as censored. Subdistribution hazard ratios were estimated using the Fine–Gray model.*

*Low physical performance was defined as a five-time chair stand test time ≥ 12 seconds. Sarcopenia was defined according to the Japan Society of Hepatology criteria (low handgrip strength and low skeletal muscle mass index).*

*CI, confidence interval; HCC, hepatocellular carcinoma; HR, hazard ratio; mALBI, modified albumin-bilirubin.*
