# Supplementary material for: The Five‐Time Chair Stand Test Identifies Patients With Liver Cirrhosis at High Mortality Risk Overlooked by Conventional Sarcopenia Criteria
Source: Int J Hepatol. 2026 Sep 6;2026:2748243. doi: 10.1155/ijh/2748243 (PMC13547947; doi:10.1155/ijh/2748243)
Supplement: Supplementary file 2 [file IJH-2026-2748243-s004.docx]

**STROBE Statement — Checklist of items that should be included in reports of cohort studies**

*Manuscript: "The five-time chair stand test identifies patients with liver cirrhosis at high mortality risk overlooked by conventional sarcopenia criteria: A Retrospective Cohort Study" (submission to International Journal of Hepatology). Page numbers refer to the revised, de-identified manuscript file (Main_document_5tcst_0727_revised.docx) as paginated for submission. This revision incorporates the title-page design descriptor, follow-up ascertainment and bias statements, missing-data/censoring statements, and relocation of the non-participation reason, addressing items 1a, 6a, 9, 12c, 12d, and 13b of the previous checklist version.*

| **Item No.** | **Recommendation** | **Reported on page / section** | **Notes for authors** |
| --- | --- | --- | --- |
| **Title and abstract** | | | |
| 1a | Indicate the study's design with a commonly used term in the title or the abstract. | Title, p.1 ("…: A Retrospective Cohort Study"); also Abstract, p.2 ("We retrospectively analyzed 241 patients…"). | — |
| 1b | Provide in the abstract an informative and balanced summary of what was done and what was found. | Abstract, p.2. | — |
| **Introduction** | | | |
| 2 | Background/rationale — Explain the scientific background and rationale for the investigation being reported. | Introduction, p.3, paragraphs 1–2. | — |
| 3 | Objectives — State specific objectives, including any prespecified hypotheses. | Introduction, p.3, final paragraph (hypothesis and two study aims). | — |
| **Methods** | | | |
| 4 | Study design — Present key elements of study design early in the paper. | Methods 2.1, p.4, first sentence ("This retrospective study…"). | — |
| 5 | Setting — Describe the setting, locations, and relevant dates, including periods of recruitment, exposure, follow-up, and data collection. | Methods 2.1, p.4 (Nagasaki University Hospital; June 2021–April 2025). | *Recruitment/data-collection dates are given, but the follow-up period and how the follow-up end-date/data-collection cut-off was defined are not explicitly stated. Consider adding one sentence on this.* |
| 6a | Participants — Give the eligibility criteria, and the sources and methods of selection of participants. Describe methods of follow-up. | Methods 2.1, p.4 (294 admitted patients; exclusion of 10 with other cancers/active infection/psychiatric disease; 43 without 5T-CST → 241 analyzed; vital status/date of death ascertained via electronic medical records, p.4). | *The follow-up ascertainment sentence added on p.4 is a draft based on standard single-center practice (currently highlighted in the manuscript). Please confirm it matches how vital status was actually determined in this cohort (e.g., whether registry or telephone confirmation was also used) before removing the highlight and submitting.* |
| 6b | Cohort study — For matched studies, give matching criteria and number of exposed and unexposed. | Not applicable. | *No matching was performed; state "not applicable" explicitly if a reviewer raises this item.* |
| 7 | Variables — Clearly define all outcomes, exposures, predictors, potential confounders, and effect modifiers. Give diagnostic criteria, if applicable. | Methods 2.1–2.3, p.4–5 (sarcopenia/handgrip/SMI/5T-CST definitions and cut-offs); confounders (age, sex, mALBI, HCC) listed in 2.4, p.5. | *The outcome (overall mortality) and how/when it was ascertained (e.g., date and cause-agnostic death record) is not explicitly defined as a separate "Outcome" subsection. Consider adding an explicit outcome definition sentence.* |
| 8* | Data sources/measurement — For each variable of interest, give sources of data and details of methods of assessment. Describe comparability of assessment methods if more than one group. | Methods 2.1–2.3, p.4–5 (medical records; Smedley dynamometer for handgrip; BIA for SMI; standardized 5T-CST protocol). | — |
| 9 | Bias — Describe any efforts to address potential sources of bias. | Methods 2.1, p.4 (statement that patients unable to complete the 5T-CST were not included, and that this may under-represent the most physically impaired patients — a potential source of selection bias); further discussed in Discussion/Limitations, p.10. | — |
| 10 | Study size — Explain how the study size was arrived at. | Methods 2.1, p.4 (all consecutive eligible admissions during the study period who completed the 5T-CST: 241/284). | *No formal sample-size/power calculation is reported; this is common for retrospective convenience cohorts, but a one-line statement that the sample size was determined by the number of eligible patients seen during the study period (not by a priori power calculation) would satisfy this item explicitly.* |
| 11 | Quantitative variables — Explain how quantitative variables were handled in the analyses; describe groupings chosen and why. | Methods 2.3, p.5 (5T-CST dichotomized at the AWGS 2019 cut-off of ≥12 s; also stratified into 4 categories: ≤9, 10–11, 12–15, ≥16 s, with rationale for the 15-s threshold cited). | — |
| 12a | Statistical methods (a) — Describe all statistical methods, including those used to control for confounding. | Methods 2.4, p.5 (Cox model; a priori covariates age, sex, mALBI grade, HCC). | — |
| 12b | Statistical methods (b) — Describe methods used to examine subgroups and interactions. | Methods 2.4, p.5–6 (Kruskal–Wallis/chi-squared across the 4 handgrip×5T-CST groups); Results 3.3–3.4, p.7–8 (Table 2, subgroup with normal handgrip strength). | *No formal interaction term (e.g., handgrip strength × 5T-CST) was tested in the Cox model; the subgroup analysis is descriptive/stratified. Consider stating explicitly that no formal interaction test was performed, or add one.* |
| 12c | Statistical methods (c) — Explain how missing data were addressed. | Methods 2.4, p.6 ("Patients with missing Child–Pugh grade or follow-up data [n = 4] were excluded from the relevant analyses [complete-case analysis]"); consistent with Table 1 (p.17) and Table 2 (p.18) footnotes. | — |
| 12d | Statistical methods (d) — Cohort study: if applicable, explain how loss to follow-up was addressed. | Methods 2.4, p.6 ("For the Kaplan–Meier and Cox regression analyses, patients alive at the time of last follow-up were censored at that date"); median follow-up (236 days) reported in Results 3.1, p.6. | — |
| 12e | Statistical methods (e) — Describe any sensitivity analyses. | Not reported. | *No sensitivity analyses (e.g., excluding HCC patients, alternative cut-offs, competing-risk analysis) are described. Optional, but would strengthen the Methods/Results if space allows.* |
| **Results** | | | |
| 13a | Participants (a) — Report numbers of individuals at each stage of the study. | Methods 2.1, p.4 (294 → 284 eligible → 241 analyzed); Supplementary Fig. S1 legend, p.12. | — |
| 13b | Participants (b) — Give reasons for non-participation at each stage. | Methods 2.1, p.4 (10 excluded for other cancer/active infection/psychiatric disease; reason the remaining 43 did not undergo the 5T-CST — omission from routine assessment or severe mobility limitations — now stated at first mention, not only in Limitations, p.10). | — |
| 13c | Participants (c) — Consider use of a flow diagram. | Referenced as Supplementary Fig. S1 (cited on p.4; legend on p.12). | *The flow diagram itself is supplementary and was not included among the files reviewed here — confirm it is uploaded with the submission package.* |
| 14a | Descriptive data (a) — Give characteristics of participants and information on exposures/confounders. | Table 1, p.17; Results 3.1, p.6. | — |
| 14b | Descriptive data (b) — Indicate the number of participants with missing data for each variable of interest. | Table 1 footnote, p.17 (Child–Pugh grade missing, n = 4); Table 2 footnote, p.18. | *Only Child–Pugh grade is flagged as missing; confirm whether any other variables (e.g., laboratory values) had missing data and, if so, report the counts.* |
| 14c | Descriptive data (c) — Cohort study: summarize follow-up time. | Results 3.1, p.6 ("median follow-up of 236 days [IQR 84–548]"). | — |
| 15* | Outcome data — Cohort study: report numbers of outcome events or summary measures over time. | Results 3.1, p.6 and Table 1, p.17 (46 deaths, 19.1%, during follow-up). | — |
| 16a | Main results (a) — Give unadjusted and confounder-adjusted estimates and their precision; state which confounders were adjusted for and why. | Table 3, p.19; Results 3.5, p.7–8 (unadjusted and adjusted HRs with 95% CI for age, sex, mALBI grade, HCC, and low physical performance). | — |
| 16b | Main results (b) — Report category boundaries when continuous variables were categorized. | Methods 2.3, p.5 (≥12 s cut-off; 4 categories: ≤9, 10–11, 12–15, ≥16 s). | — |
| 16c | Main results (c) — If relevant, translate relative risk into absolute risk for a meaningful time period. | Partially: crude mortality rates given as percentages (29.6% vs 12.1%), Results 3.4, p.7. | *Only crude percentages, not risk over a defined time period (e.g., 1-year mortality) or absolute risk difference, are reported. Optional enhancement given the short/variable follow-up.* |
| 17 | Other analyses — Report other analyses done (subgroups, interactions, sensitivity analyses). | Table 2 and Results 3.3, p.7 (4-group analysis by handgrip strength × 5T-CST status); Fig. 1 (trend analyses, p.6–7); Fig. 2 and Supplementary Fig. S2 (Kaplan–Meier analyses, p.7 and p.12). | — |
| **Discussion** | | | |
| 18 | Key results — Summarize key results with reference to study objectives. | Discussion, p.8, first paragraph; Conclusion, p.10. | — |
| 19 | Limitations — Discuss limitations, taking into account sources of potential bias or imprecision, and their direction/magnitude. | Discussion, p.10 (single-center design, incomplete 5T-CST performance/selection bias, small discordant subgroup, missing cause-of-death data, limited events restricting covariates). | *Limitations are well described qualitatively; the direction/likely magnitude of each potential bias (e.g., whether selection bias would over- or under-estimate the association) is not explicitly discussed. Consider adding one sentence per major bias on its likely direction.* |
| 20 | Interpretation — Give a cautious, overall interpretation considering objectives, limitations, multiplicity of analyses, and other evidence. | Discussion, p.8–9 (comparison with AWGS 2025 framework and prior 5T-CST/muscle-function literature). | — |
| 21 | Generalisability — Discuss the generalisability (external validity) of the study results. | Discussion, p.10 ("single-center retrospective study, which may limit the generalizability of the findings"). | — |
| **Other information** | | | |
| 22 | Funding — Give the source of funding and the role of funders for the present study and, if applicable, the original study on which the article is based. | Declarations, p.11 ("received no specific grants from any funding agency…"). | — |

** Give information separately for exposed and unexposed groups where applicable (item denoted with an asterisk in the original STROBE checklist).*

Note: this checklist is based on the STROBE Statement (von Elm E, Altman DG, Egger M, Pocock SJ, Gøtzsche PC, Vandenbroucke JP; STROBE Initiative. The Strengthening the Reporting of Observational Studies in Epidemiology (STROBE) statement: guidelines for reporting observational studies. Lancet. 2007;370:1453–7), available at www.strobe-statement.org. An explanation and elaboration article discusses each checklist item and gives methodological background and published examples of transparent reporting. The STROBE checklist is best used in conjunction with this article (freely available on the websites of PLoS Medicine, Annals of Internal Medicine, and Epidemiology). Information on the STROBE Initiative is available at www.strobe-statement.org.
